# Bacteria and Mold Spore Heat Resistance in Guava Juice and Its Control by pH and Sodium Benzoate

**DOI:** 10.1155/2021/5594362

**Published:** 2021-06-15

**Authors:** Evelyn, Chairul

**Affiliations:** Department of Chemical Engineering, University of Riau, Pekanbaru 28293, Indonesia

## Abstract

Heat-resistant bacteria and molds can survive the pasteurization conditions used in high-acid fruit juices. The objective of this study was to evaluate the log reductions and thermal inactivation kinetics of spores of *Bacillus subtilis* bacteria and ascospores of *Talaromyces flavus* and *Eupenicillium javanicum* molds under influence of pH and sodium benzoate preservative. The spores were suspended in guava juice, processed at 90-100°C for *B. subtilis* and at 80-90°C for *T. flavus* and *E. javanicum*, and decimal reduction (*D*) values were estimated from the log survivor curves. Next, the effects of pH change (3.5-4.5) and 0.015% sodium benzoate addition on the *D* values of spores were investigated. Lower *D* values were obtained at higher temperatures (*D*_100°C_ value of 2.32 min vs. *D*_90°C_ value of 15.33 min for *B. subtilis*, *D*_90°C_ value of 2.96 min vs. *D*_80°C_ value of 59.52 min for *T. flavus*, and *D*_90°C_ value of 1.58 min vs. *D*_80°C_ value of 21.32 min for *E. javanicum*). The *D* values decreased further (to 1.8 min at 100°C for *B. subtilis*, to 2.33 min at 90°C for *T. flavus*, and to 1.49 min at 90°C for *E. javanicum*) when the pH of guava juice was decreased from 4.1 to 3.5. Inclusion of sodium benzoate in pH 3.5 juice enhanced the thermal inactivation of spores (*D*_100°C_ value decreased to 1.4 min for *B. subtilis*, to 1.98 min for *T. flavus*, and to 1.34 min for *E. javanicum*). To conclude, the combination of low pH and sodium benzoate provided the best method for spore inactivation, which could enhance food safety and extend food's shelf life.

## 1. Introduction

*Talaromyces* sp. and *Eupenicillium* sp. have been listed as heat-resistant molds due to their ability to form ascospores. Both molds are widely distributed in the soil, making fruit products susceptible to these mold contaminations. Ascospore germination and growth in fruit products after pasteurization reaching commonly 10^5^-10^6^/g or mL in number are a concern since it can cause food spoilage and in some cases foodborne diseases. Decimal reduction values (*D* values) at 91°C between 0.9 min in strawberry pulp and 2.9 min in grape juice have been reported for *Talaromyces* sp. ascospores [1, 2]. With respect to *Eupenicillium* sp. ascospores, previous studies have shown that the *D* values in strawberry pulp and pineapple juice were 15-19.8 min, 3.7-5.0 min, and 0.8-1.5 min at 80°C, 85°C, and 90°C, respectively [1, 3]. *Bacillus* sp., such as *Bacillus subtilis*, is also one of the predominant microbes from bacterial genera found in soil and may also be introduced into the manufacturing process through poorly washed fruits. *B. subtilis* is one of the most heat-resistant bacteria, resembling *D*_95°C_ value of 15.8 min and *D*_100°C_ value of 5.7 min in tomato juice [4]. Our recent results for this bacterium showed the *D* value of 6.75 min at 95°C and 2.11 min at 100°C in pineapple juice [5]. No other reports were reported for this bacterium spores in high-acid foods.

Some tropical countries are rich in the production of exotic fruits such as mango, orange, pineapple, guava, durian, and avocado. The abundant production and high nutritional contents of these fruits have made them processed into various juice blends. Guava (*Psidium guava* L.) is one of the very appreciated and preferred consumed juices due to its unique banana-like aroma and delicate flavour. Contamination and isolation of molds such as *Talaromyces flavus* and *Eupenicillium javanicum* and bacteria such as *Bacillus subtilis* in juices have been reported in past literatures [6–10]. Thus, a proper preservation method should be attempted to avoid significant economic losses and maintain food safety as well as sensorial and quality.

Various types of preservatives have been investigated in past studies to investigate its effect on the sensitivity of microorganisms in food products and to extend food's shelf life. The preservatives reported were sorbic acid, benzoic acid, potassium sorbate, and sodium sulfite or nitrite [11], in which some of them have often been used in the food industry. Several factors such as pH and composition of the food product and the type of microorganism determine the inhibitory effect of the preservatives. Benzoic acid in the form of sodium benzoate is an antibacterial and antifungal agent and is widely used to preserve margarine, fresh juices, and sweets in the food industry. Common ranges of sodium benzoate used in foods are between 0.01% and 0.1% [10], with a limit of up to 0.5% were reported by European Commission [12].

Thermal processing is still the most widely applied technology in the food industry to preserve juices and beverages through inactivation of microorganisms. Typical juice processing temperatures of 95°C for 2 min may not sufficiently reduce the spores of heat-resistant microorganisms leading to juice spoilage. Determining suitable conditions to process a certain juice contaminated by certain microorganisms and investigating the potential enhancement of heat in combination with other preservation factors, among them antimicrobials, are necessary. Therefore, due to the importance of *T. flavus* and *E. javanicum* molds and *B. subtilis* bacteria in fruit juices, the objectives of this study were to investigate the heat, pH, and sodium benzoate sensitivity of these bacterial and mold spores in guava juice, followed by the determination of the first-order kinetic parameters of these bacterial and mold spores in guava juice after the thermal inactivation.

## 2. Materials and Methods

### 2.1. Heat-Resistant Molds and Bacteria

*Talaromyces flavus* InaCC F155, *Eupenicillium javanicum* InaCC F154, and *Bacillus subtilis* B1204 were purchased from Indonesian Culture Collection (InaCC). Both molds were sourced from Indonesian soil, whereas *B. subtilis* was isolated from Curcuma zedoaria (stem). All microorganisms were revived according to the suppliers' instructions.

### 2.2. Spore Production

Ascospores of *T. flavus* and *E. javanicum* were obtained according to previous methods [13, 14]. Briefly, the spore formation for both molds was confirmed after growth for four weeks at 30°C for periods of 30 days on potato dextrose agar (PDA), whereas the spore formation for *B. subtilis* was confirmed after incubation at 37°C for 14 days on nutrient agar. The final spore suspension was then stored at 2°C in SDW.

### 2.3. Guava Juice Preparation and Inoculation

Commercial guava juices were obtained from a local supermarket. The juices have a soluble solid content of 11.4 ± 0.1°Brix and pH 4.1 ± 0.1 and were used as the heating medium for *T. flavus*, *E. javanicum*, and *B. subtilis* spores. Aliquots (ca. 1.0 mL) of mold or bacterial suspension were inoculated into 9.0 mL of guava juice to yield an initial spore concentration of approximately 10^8^ or 10^9^ cfu/mL of juice. NaOH and HCl were used to adjust the pH of guava juices to 3.5 and 4.5 (determined with a digital pH meter, Mettler Toledo, USA) since these are common range pH values of fruit juices. Sodium benzoate was obtained from a local supermarket and added to the pH 3.5 juice at a concentration of 0.015% (*w*/*v*) in thermal processing with sodium benzoate. For each temperature, 0.075 g of sodium benzoate was added into 500 mL of guava juice.

### 2.4. Thermal Processing

Thermal treatments were carried out according to previous methods [13, 14]. For *T. flavus* and *E. javanicum* ascospores, thermal death tubes containing the inoculated guava juices were immersed in preheated thermostatic water bath at 80, 85, and 90°C for different time intervals (up to 50 min with every 5 min). For *B. subtilis*, heating was performed at 90, 95, and 100°C (up to 40 min with every 5 min). Higher temperatures were attempted for *B. subtilis* since bacteria is generally more heat resistant than molds. After thermal treatment, the tubes were immediately transferred to an ice bath until microbial enumeration. Each experiment was carried out twice.

### 2.5. Spore Enumeration

The spore concentration in guava juice before and after processing was determined by spread plating onto PDA for the molds and onto nutrient agar for the bacteria. Prior to plating, 1 mL spore samples were decimal diluted using 9 mL of 0.85% saline solution for molds [13, 14], while 9 mL of 0.1% (*w*/*v*) sterile buffered peptone water was used for bacteria. Each tube dilution was mixed repeatedly using a high-speed vortex mixer to yield a uniform spore suspension and plated twice. The plates for bacteria were then incubated at 37°C for 24-48 hr, whereas the plates for molds were incubated for 3 to 5 days until visible colonies were seen. Average colony counts were calculated and spore concentration was expressed in cfu/mL of juice sample.

### 2.6. Data Model Fitting and Statistical Analysis

The spore logarithmic reductions (log *N*/*N*_0_) after the thermal treatments were calculated and plotted using Microsoft Excel 2013 (Microsoft Inc., USA). Then, the first order *D* and *z* value thermal resistance parameters were estimated based on the survival curves for the mold and bacterial species [5, 14, 15]. The coefficient of determination (*R*^2^) was used to compare the goodness of fit of the model. *N*_0_ is the initial or untreated ascospore population (cfu/mL), and *N* is the number of ascospores after being exposed to a lethal (heat) treatment for a specific time (*t*). *D* or *D*_*T*_ values are decimal reduction times, which is the time in min at a certain temperature necessary to reduce the microbial population by 90%), whereas *z* is temperature coefficient in °C which is a temperature increase that results in a 10-fold decrease in the *D*_*T*_ value [16]. The *D*_*T*_ values were calculated from the reciprocal of the slope in Equation (1) and *z*_*T*_ values were estimated from the negative reciprocal of the slope as in Equation (2). *D*_*T*ref_ is the *D* value at the reference temperature or *T*_ref_ (can be any reference temperature, °C) and *T* is the temperature of the isothermal treatment.

(1) $$\begin{array}{c} \log\frac{N_{\left(t\right)}}{N_{0}}=-\frac{t}{D_{T}}, \end{array}$$

(2) $$\begin{array}{c} \log\left(\frac{D_{T}}{D_{T\text{ref}}}\right)=\left(\frac{T_{\text{ref}}-T}{z_{T}}\right). \end{array}$$

Microsoft Excel 2013 was also used for statistical analysis of data. Single-factor ANOVA was performed to analyze synergistic effects of pH and sodium benzoate on the *D* values of the same strain at 80-90°C using confidence level of 95%.

## 3. Results and Discussion

### 3.1. Heat Sensitivity of Bacterial and Mold Spores in Guava Juice

**Figure 1:**
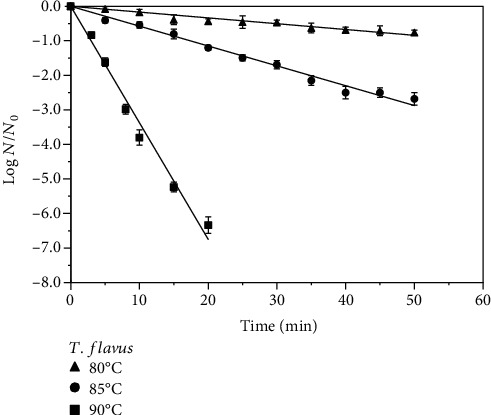
Heat inactivation of *Talaromyces flavus* ascospores in guava juice (11.4°Brix, pH 4.1) at 80°C-90°C.

**Figure 2:**
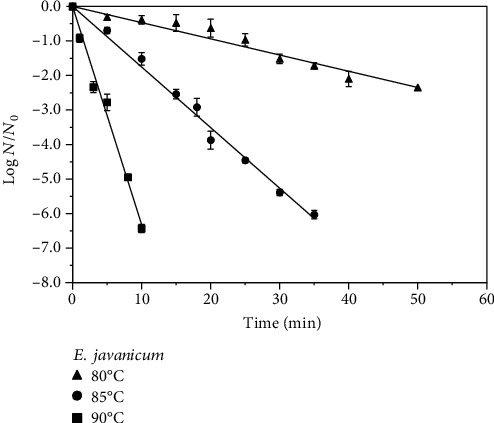
Heat inactivation of *Eupenicillium javanicum* ascospores in guava juice (11.4°Brix, pH 4.1) at 80°C-90°C.

**Figure 3:**
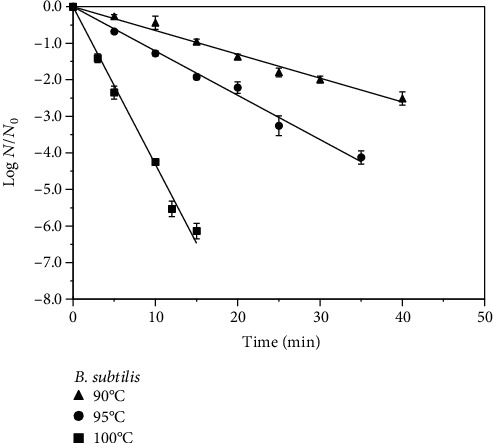
Heat inactivation of *Bacillus subtilis* spores in guava juice (11.4°Brix, pH 4.1) at 90°C-100°C.

The log reduction profiles of ascospores of *T. flavus*, *E. javanicum*, and *B. subtilis* in guava juice (pH 4.1 and 11.4°Brix) obtained after heating at 80-90°C are shown in Figures 1–3. As expected, higher log reductions of the spores were obtained at higher temperatures for the same processing time. For example, a 10°C rise in the temperature from 80°C to 90°C for 20 min resulted in the increase of *T. flavus* inactivation in guava juice by 5.9 log. For *E. javanicum*, the same process for 10 min resulted in 6.3 log reduction of these spores. With respect to *B. subtilis*, increasing the temperature from 90°C to 100°C for 10 min increased the inactivation by 3.7 log. Except for *E. javanicum*, we previously reported 6.5 log reduction for *T. flavus* and 4.6 log reduction for *B. subtilis* in pineapple juice [5, 13], in which generally showed higher resistance in this study with guava juice for the same process conditions.

Based on the linear appearance of the log reduction profiles in Figures 1–3, decimal reduction time (*D*) values were derived from the linear regressions of the best-fit straight line. The temperature changes that produced a 10-fold change in the *D* values were also obtained and both are presented in Table 1. In general, the first-order kinetic models are supported by the *D* value temperature dependence (*R*^2^ = 0.94 − 0.99). The increase in the heat sensitivity of spores with increasing temperature can be shown by lower *D* values at higher temperatures. For *T. flavus*, the *D* value obtained at 90°C was 2.96 ± 0.06 min as opposed to 17.39 ± 0.75 min at 85°C and 59.52 ± 1.24 min at 80°C. Regarding *E. javanicum*, the *D* value found was 1.58 ± 0.04 min at 90°C vs. 5.70 ± 0.17 min at 85°C and 21.32 ± 0.90 min at 80°C. *B. subtilis* showed higher resistance in guava juice with the *D* values obtained being 15.33 ± 1.7 min, 8.25 ± 0.9 min, and 2.32 ± 0.7 min at 90°C, 95°C, and 100°C, respectively, as bacteria commonly exhibit higher resistance than mold or yeast spores. Past studies showed the *D*_90°C_ values of these bacteria and mold spores in pineapple juices which were slightly lower than reported in this study, i.e., 2.89 min for *T. flavus* [13], 1.45 min for *E. javanicum* [3], and 13.2 min for *B. subtilis* [4], confirming the food matrix effect. Nonetheless, the obtained *D*_90°C_ values are still in the common range reported values in various juices for these molds and bacteria in past studies (1.13-7.5 min) [1, 3, 13, 17–19]. It has been known that differences in the *D* values of bacterial and mold spores are not only affected by strain but also by many other factors such as the composition of fruit products and medium or sporulation conditions prior to heat treatment. The *z* values estimated in this case were between 7.7°C and 8.9°C for molds and 12.2°C for *B. subtilis* (Table 1), which are within the expected values for these microorganisms. The *z* values also reflected the heat resistance of microbes, being higher for the bacteria than for the molds. To conclude, 95°C-2 min of fruit juice processing would remove the spores of *E. javanicum*; however, it is not suitable for a 5-log reduction of *T. flavus* and *B. subtilis* spores.

### 3.2. pH Sensitivity of Bacterial and Mold Spores in Guava Juice

Table 2 shows the effect of pH change on the decimal reduction (*D*) values of spores of *T. flavus and E. javanicum* molds and *B. subtilis* bacteria in guava juices adjusted to pH 3.5 and 4.5. Generally, the microorganisms tend to show greater heat sensitivity at lower juice's pH resulting in lower *D* values. For *T. flavus* in pH 3.5 juice, the *D*_*T*_ values obtained to inactivate 1-log of spores were 48.21 ± 1.15 min at 80°C, 15.82 ± 0.52 min at 85°C, and 2.33 ± 0.02 min at 90°C. Meanwhile, in pH 4.5 juice, the *D*_*T*_ values found to inactivate 1-log of spores were 71.42 ± 1.69 min at 80°C, 27.59 ± 1.10 min at 85°C, and 2.86 ± 0.05 min at 90°C. For *E. javanicum* in pH 3.5 juice, the *D*_*T*_ values found for 1-log of spores were 17.70 ± 0.65 min at 80°C, 5.13 ± 0.12 min at 85°C and 1.49 ± 0.04 min at 90°C. Meanwhile, for pH 4.5, the *D*_T_ values found 1-log of spores were 26.65 ± 0.94 min at 80°C, 8.35 ± 0.22 min at 85°C, and 1.68 ± 0.03 min at 90°C. The greater sensitivity of microorganisms in lower pH juices was also confirmed for *B. subtilis.* For example, the *D*_90°C_ values of 11.2 ± 0.23 min vs. 17.9 ± 0.42 min were obtained at pH 3.5 and pH 4.5, respectively. In addition, the *D*_100°C_ value of 1.8 ± 0.02 min was obtained at pH 3.5 and the *D*_100°C_ value of 2.5 ± 0.01 min was obtained at pH 4.5, although the differences were small. Past studies have shown that the heat resistance of spores of bacteria from *Bacillus* and *Clostridium* genera increased with increasing pH values and displayed maximum values at pH close to neutrality [20–22]. Similar observations of decreasing sensitivity of spores with pH values have also been observed by other investigators with *Aspergillus flavus* and *Penicillium digitatum* mold spores (pH 3.0 to 5.5) and *Alicyclobacillus acidoterrestris* spores (pH 2.5 to 6.0) [23, 24]. Although the *z* values were slightly lower in higher pH juices for all microorganisms, these observations were in agreement with the results of *A. acidoterrestris* spore thermal inactivation in grapefruit juices with pH change from 3.0 to 4.0 [25].

### 3.3. Sodium Benzoate Sensitivity of Bacterial and Mold Spores in Guava Juice

Sensitivity of *T. flavus and E. javanicum* molds and *B. subtilis* bacteria to sodium benzoate indicated by the decimal reduction (*D*) values in guava juice at pH 3.5 was studied, and the results are presented in Table 3. Generally, the molds exhibited greater heat sensitivity than the bacterial spores. As can be seen from Table 3, the addition of 0.015% sodium benzoate caused further decreases in the *D* values of all tested microbial spores in guava juices, with a maximum reduction value of around 30% being observed for *T. flavus*. The *D*_*T*_ values obtained for *T. flavus* were 33.33 ± 1.12 min at 80°C, 10.43 ± 0.15 min at 85°C, and 1.98 ± 0.04 min at 90°C. Meanwhile, for *E. javanicum*, the *D*_*T*_ values achieved were 12.80 ± 0.60 min at 80°C, 3.59 ± 0.03 min at 85°C, and 1.34 ± 0.01 min at 90°C. With respect to *B. subtilis*, the *D* values found were 10.9 ± 0.33 min, 5.2 ± 0.05 min, and 1.4 ± 0.02 min at 80°C, 85°C, and 90°C, respectively.

**Figure 4:**
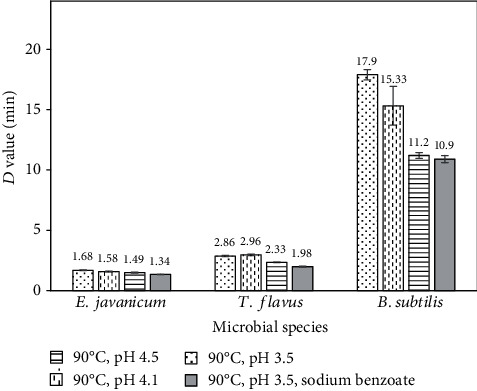
Comparison of decimal reduction values of *Bacillus subtilis* bacteria and *Talaromyces flavus* and *Eupenicillium javanicum* molds in 11.4°Brix guava juice at 90°C (values are average ± standard deviation).

Bar charts for the *D* values between 80 and 90°C were further constructed, and the results at 90°C are presented in Figure 4. Then, statistical analysis was carried out to compare the difference in the *D* values. Analysis results showed that there were significant differences in the *D* values of the same strain after all treatments (*p* < 0.05) and more prone at lower temperatures, suggesting a synergistic effect between low pH and sodium benzoate to reduce the heat resistance of these microorganisms. The inactivation effect of heat combined pH 3.5-sodium benzoate was better than the inactivation effect of heating at pH 3.5 alone or higher pHs, i.e., pH 4.1 and 4.5. According to Restaino et al. [26], lowering pH of the medium and increasing the concentration or addition of preservatives would inhibit the growth and activity of microorganisms. However, the effectiveness of sodium benzoate to sensitize microorganisms decreased at higher pH, which is in agreement with other studies [27]. Reductions in the *D* values of several molds (*B. nivea*, *Aspergillus flavus*, *Penicillium puberulum*, and *Geotrichum candidum*) after addition of 1000 *μ*g/mL of sodium benzoate have been observed in past studies [28]. Inhibitory effects of sodium benzoate on heat-damaged *Bacillus stearothermophilus* spores have also been reported by other authors [29]; however, to the best of our knowledge, the heat sensitization of spores of *B. subtilis* by sodium benzoate was first reported in this study. Nevertheless, the effectiveness of chemical preservatives is often limited by their actions or adverse effects in food products [30].

## 4. Conclusion

*Bacillus subtilis* had higher heat resistibility in the guava juice compared to the molds (*Talaromyces flavus* and *Eupenicillium javanicum*). The spores of *B. subtilis* decreased by 5-log cycles within 11.6 min at 100°C whereas 3.61 min were required for *T. flavus* at 95°C, which exceeds the typical juice processing conditions. The logarithmic values of surviving spores of these microorganisms in the juice followed the first-order kinetic model with coefficient determinations of more than 0.93. Lowering the pH of the juice and inclusion of 0.015% sodium benzoate in the juice prior to heating increased the bacteria and molds' heat sensitivity and thus decreased the resistance to pasteurization.

## Figures and Tables

**Table 1:** Heat resistance of spores of *Talaromyces flavus* and *Eupenicillium javanicum* molds and *Bacillus subtilis* bacteria in guava juice (pH 4.1).

Species	Temperature (°C)	*D*_*T*_ value (min) ± SD^∗^	*z* value (°C) ± SE
Mold:	80	59.52 ± 1.24	7.7 ± 0.10

*T. flavus*	85	17.39 ± 0.75	*R* ^2^ = 0.94 − 0.99
	90	2.96 ± 0.06	

Mold:	80	21.32 ± 0.90	8.9 ± 0.01

*E. javanicum*	85	5.70 ± 0.17	*R* ^2^ = 0.94 − 0.99
	90	1.58 ± 0.04	

Bacteria:	90	15.33 ± 1.7	12.2 ± 0.07

*B. subtilis*	95	8.25 ± 0.9	*R* ^2^ = 0.94 − 0.97
	100	2.32 ± 0.7	

^∗^ *D* values shown are average of two experiments ± standard deviation.

**Table 2:** The effect of pH change on the heat resistance of spores of *Talaromyces flavus* and *Eupenicillium javanicum* molds and *Bacillus subtilis* bacteria in guava juice.

Species	pH	Temperature (°C)	*D*_*T*_ value (min) ± SD^∗^	*z* value (°C) ± SE
Mold:	3.5	80	48.21 ± 1.15	7.6 ± 0.14

*T. flavus*		85	15.82 ± 0.52	*R* ^2^ = 0.94 − 0.99
		90	2.33 ± 0.02	
	4.5	80	71.42 ± 1.69	7.2 ± 0.23
		85	27.59 ± 1.10	*R* ^2^ = 0.93 − 0.98
		90	2.86 ± 0.05	

Mold:	3.5	80	17.70 ± 0.65	9.3 ± 0.01

*E. javanicum*		85	5.13 ± 0.12	*R* ^2^ = 0.96 − 0.99
		90	1.49 ± 0.04	
	4.5	80	26.65 ± 0.94	8.3 ± 0.08
		85	8.35 ± 0.22	*R* ^2^ = 0.96 − 0.99
		90	1.68 ± 0.03	

Bacteria:	3.5	90	11.2 ± 0.23	12.6 ± 0.12

*B. subtilis*		95	6.3 ± 0.11	*R* ^2^ = 0.94 − 0.99
		100	1.8 ± 0.02	
	4.5	90	17.9 ± 0.42	11.7 ± 0.18
		95	10.2 ± 0.16	*R* ^2^ = 0.93 − 0.98
		100	2.5 ± 0.01	

^∗^ *D* values shown are average of two experiments ± standard deviation.

**Table 3:** Heat resistance of spores of *Talaromyces flavus* and *Eupenicillium javanicum* in guava juice (pH 3.5) treated with 0.015% sodium benzoate.

Species	Temperature (°C)	*D*_*T*_ value (min) ± SD^∗^	*z* value (°C) ± SE
Mold:	80	33.33 ± 1.12	8.2 ± 0.05

*T. flavus*	85	10.43 ± 0.15	*R* ^2^ = 0.95 − 0.99
	90	1.98 ± 0.04	

Mold:	80	12.80 ± 0.60	10.2 ± 0.05

*E. javanicum*	85	3.59 ± 0.03	*R* ^2^ = 0.95 − 0.99
	90	1.34 ± 0.01	

Bacteria:	90	10.9 ± 0.33	11.2 ± 0.13

*B. subtilis*	95	5.2 ± 0.05	*R* ^2^ = 0.93 − 0.98
	100	1.4 ± 0.02	

^∗^ *D* values shown are average of two experiments ± standard deviation.

## Data Availability

All the data relevant to the research can be found in the manuscript. Any further information is available from the corresponding author upon request.
